# Developmental Dyslexia: Insights from EEG-Based Findings and Molecular Signatures—A Pilot Study

**DOI:** 10.3390/brainsci14020139

**Published:** 2024-01-28

**Authors:** Daniela Theodoridou, Christos-Orestis Tsiantis, Angeliki-Maria Vlaikou, Vasiliki Chondrou, Victoria Zakopoulou, Pavlos Christodoulides, Emmanouil D. Oikonomou, Katerina D. Tzimourta, Charilaos Kostoulas, Alexandros T. Tzallas, Konstantinos I. Tsamis, Dimitrios Peschos, Argyro Sgourou, Michaela D. Filiou, Maria Syrrou

**Affiliations:** 1 Laboratory of Biology, Faculty of Medicine, School of Health Sciences, University of Ioannina, 45110 Ioannina, Greecepmd00125@uoi.gr (C.-O.T.); 2 Biomedical Research Institute, Foundation for Research and Technology-Hellas (FORTH), 45110 Ioannina, Greecemfiliou@uoi.gr (M.D.F.); 3 Laboratory of Biochemistry, Department of Biological Applications and Technology, School of Health Sciences, University of Ioannina, 45110 Ioannina, Greece; 4 Laboratory of Biology, School of Science and Technology, Hellenic Open University, 26335 Patras, Greece; vchondrou@eap.gr (V.C.); sgourou@eap.gr (A.S.); 5 Department of Speech and Language Therapy, School of Health Sciences, University of Ioannina, 45110 Ioannina, Greece; vzakop@uoi.gr (V.Z.); pchristo@uoi.gr (P.C.); 6 Laboratory of Physiology, Faculty of Medicine, School of Health Sciences, University of Ioannina, 45110 Ioannina, Greece; ktsamis@uoi.gr (K.I.T.); dpeschos@uoi.gr (D.P.); 7 Department of Informatics and Telecommunications, School of Informatics & Telecommunications, University of Ioannina, 47100 Arta, Greece; e.oikonomou@uoi.gr (E.D.O.); tzallas@uoi.gr (A.T.T.); 8 Department of Electrical and Computer Engineering, University of Western Macedonia, 50100 Kozani, Greece; 9 Laboratory of Medical Genetics, Faculty of Medicine, School of Health Sciences, University of Ioannina, 45110 Ioannina, Greece

**Keywords:** stress, developmental dyslexia, electroencephalogram, HPA axis genes

## Abstract

Developmental dyslexia (DD) is a learning disorder. Although risk genes have been identified, environmental factors, and particularly stress arising from constant difficulties, have been associated with the occurrence of DD by affecting brain plasticity and function, especially during critical neurodevelopmental stages. In this work, electroencephalogram (EEG) findings were coupled with the genetic and epigenetic molecular signatures of individuals with DD and matched controls. Specifically, we investigated the genetic and epigenetic correlates of key stress-associated genes (*NR3C1*, *NR3C2*, *FKBP5*, *GILZ*, *SLC6A4*) with psychological characteristics (depression, anxiety, and stress) often included in DD diagnostic criteria, as well as with brain EEG findings. We paired the observed brain rhythms with the expression levels of stress-related genes, investigated the epigenetic profile of the stress regulator glucocorticoid receptor (GR) and correlated such indices with demographic findings. This study presents a new interdisciplinary approach and findings that support the idea that stress, attributed to the demands of the school environment, may act as a contributing factor in the occurrence of the DD phenotype.

## 1. Introduction

Developmental dyslexia (DD) (Although Specific Learning Disorder is the generic term used in the DSM-5 to describe disorders characterized by difficulties in learning and academic skills, we adopt the term developmental dyslexia (DD) as it is an impairment that affects the development of decoding skills in reading, which is the core focus of this study, differentiating it from the other learning disorders of dyscalculia and disorders of written expression [1]) is a phenotypically and genetically heterogeneous, multifactorial, complex condition characterized by difficulties in reading, writing and spelling, which affects 5–20% of the general population [2] and 5–17% of school-age children [3]. Adults with DD present phonological processing impairments [4,5], working memory deficits [6], primary auditory processing abnormalities, impaired visual processing [7,8], attentional deficits [9] and irregularities in the processing of written information [10]. These difficulties, usually intertwined with a constant pattern of failure, provide strong conditions for emotional and psychological issues to arise (low self-esteem, anxiety, feelings of isolation) [11]. Although diagnosed in childhood, DD is a lifelong condition, possibly intensified by adverse experiences that occur during the lifetime, with putative additive and reinforcing effects on the phenotype [12,13,14,15].

Several genes and gene variants have been associated with DD predisposition, but, as in all complex conditions, the phenotypic and genotypic heterogeneity is the result of the interplay between genetic and non-genetic environmental factors, in the form of epigenetically encoded information (gene × environment interactions, G × E) [16,17,18,19,20,21,22,23]. According to recent hypotheses and research findings, among environmental factors, stress is widely documented as a contributing factor in the etiopathogenesis of complex disorders, due to its effects on brain plasticity and function, particularly during early developmental stages that are critical for neurodevelopment [24,25,26,27].

In response to a stressor, hypothalamic–pituitary–adrenal (HPA) axis-related genes and especially glucocorticoid signaling pathway genes mediate the stress response [28]. Glucocorticoid receptor (GR), encoded by the Nuclear Receptor Subfamily 3 Group C Member 1 (*NR3C1)* gene, and mineralocorticoid receptor (MR), encoded by the Nuclear Receptor Subfamily 3 Group C Member 2 (*NR3C2*) gene, mediate the physiological stress response and the organism’s homeostasis. *NR3C1* expression levels are altered in anxiety and mood disorders and alterations have been already implicated in the DD phenotype in humans [29,30]. A methylation analysis of *NR3C1* and its alternative, non-coding first exons revealed a strong association between stress and methylation levels and the occurrence of depressive episodes after prenatal adversity and adult adverse life experiences [31,32,33]. Glucocorticoid-induced leucine zipper (*GILZ*) is a GR-induced measure of the stress response that is transcriptionally activated by glucocorticoids and links the neuroendocrine stress response to immune-related disorders [34]. FK506 Binding Protein 5 (*FKBP5)* is a co-chaperone of GR, the levels of which were found altered in complex stress-associated phenotypes [35]. The functional single nucleotide variant (SNV) rs1360780 has been implicated in the occurrence of complex phenotypes and stress-associated psychopathology, with the (T) allele considered as a risk allele [26,36,37]. Solute Carrier Family 6 Member 4 *(SLC6A4)* encodes the serotonin transporter gene. The polymorphic 5-HTTLPR region located in its promoter, which also encompasses rs25531, has been linked to an increased risk of anxiety and depression-related disorders [38,39].

Glucocorticoids have been shown to regulate mitochondrial function [40] and GR has been reported to regulate mitochondrial DNA (mtDNA) gene expression [41]. Mitochondria have recently emerged as pathophysiology hubs and potential therapeutic targets for stress-related and anxiety disorders [42,43,44,45,46,47,48].

The current diagnostic approach for DD and specific learning disorders employs standardized clinical tests and tools to assess phonological skills, reading and spelling, while neuroimaging modalities are only used to exclude other possible diagnoses [49,50]. Diagnostic tools with high accuracy in internalizing disorders are also widely used in everyday clinical practice to test the association between reading problems and anxiety or depression [51,52]. Anxiety disorders have been repeatedly associated with alterations in the electroencephalography (EEG)-recorded neuronal network oscillations [53]. Research findings, on the other hand, focus on abnormalities observed on magnetic resonance imaging (MRI), EEG, magnetoencephalography (MEG) and biometric signals, such as heart rate, electrodermal activity (EDA) and eye tracking [54,55,56,57], which enhance our understanding of the pathophysiology and could be used for DD diagnosis and management [58,59,60,61].

EEG is a low cost, non-invasive method of recording the electrical activity on the scalp, which represents the activity of the cerebral cortex [62]. A set of distinct factors extracted from the spectral analysis of EEG signals that can provide bio-evidence of dyslexia was proposed [63]. Accordingly, in a recent report studying the functional brain connections in children with and without dyslexia using resting-state EEG data, brain network deficiencies were observed when performing various spelling, phonological and morphological awareness tasks [64]. Synchronized neuronal excitation produces explicit rhythms on EEG waves that have been related to distinct cognitive states and to brain disorders [65,66]. In studies focusing on EEG rhythms’ changes, increased frontal theta and beta rhythm activity was reported in the right parietal–occipital area in children with dyslexia when performing phonological tasks, while other studies have reported greater consistency in delta, theta and beta rhythms and lower consistency in alpha frequencies during relaxation [67,68,69,70].

In adults with dyslexia, research findings have underscored low brain activation in a variety of brain regions, such as reduced activation of the left inferior angular gyrus during phonological tasks [71,72], reduced activation in the frontal network of the left hemisphere associated with phonological working memory [73] and increased activation of the left inferior frontal gyrus during phonological tests [74].

The diagnostic coupling of the EEG method with machine learning techniques has given new insights into the neuronal network deficiencies underlying specific learning disorders, providing a wealth of information for their diagnosis and pathophysiology [64,75,76,77]. Despite the absence of a consensus on the methodology for implementation in the diagnosis [60,70,78], it has been shown that recently established EEG research protocols have diagnostic relevance in DD [56,79], contributing to the classification [80] and better understanding of brain function [81]. Such techniques were also applied in a study including children with and without dyslexia, which concluded that it is possible to identify underlying neurocognitive profiles for dyslexia and therefore to distinguish between the two populations through the information extracted from EEG [82]. In studies using algorithms to interpret EEG data, the classification accuracy reached 90% (study at rest) and 100% (study during testing), respectively [83,84].

Moreover, EEG data are also used by researchers (a) as part of multifactorial approaches to identify dyslexia [85], (b) as a means of recording emotions to analyze symptoms of both dyslexia and other disorders such as autism and ADHD [86], (c) as a means of comparing brain function between “competent” children with dyslexia (capable dyslexic) and severe cases of dyslexia (poor dyslexic) [87] and (d) as a means of recording changes in cerebral laterality after an intervention period in children with dyslexia [88].

The search for reliable biomarkers in medicine is a crucial issue for the development of effective personalized treatment strategies [89]. Although validating biomarkers for neurodevelopmental conditions carries difficulties, primarily due to the heterogeneity of these complex conditions, the use of the most widely accepted modalities to date—namely non-invasive neuroimaging, genetic and epigenetic data and the correlation of the findings—emerges as a promising strategy to advance the field [90]. EEG parameters have been combined with genetic and epigenetic findings of stress-related genes to characterize some neurological conditions. In a study, the *FKBP5* rs1360780 and *FKBP5* promoter methylation status was combined with EEG parameters in Alzheimer’s disease patients [91], while, in another study, EEG was used to explore the contribution of MR gene variants in habit learning under stress [92].

This approach necessitates a multidisciplinary teamwork, encompassing the co-evaluation of established diagnostic criteria complemented by neuropsychological assessment and advanced neuroimaging techniques, as well as evidence from molecular biomarkers. This multidisciplinary study correlates the already established diagnostic criteria for DD with EEG recordings and stress-associated molecular signatures.

The DD phenotype is not solely attributed to susceptibility genes, which have been identified but are not considered the major etiologic factor. The phenotype is the result of the interaction of environmental factors (risk or protective), along with a genetic predisposition, but the relative impact of each component is not easily traceable [13,93,94]. Stress appears often in the literature as an important factor in the context of DD, and the stress experienced by DD individuals may compromise the negative feedback mechanisms, thereby leading to HPA axis dysregulation [16,17,19,27].

As in all complex, heterogeneous phenotypes, the DD phenotype results from the interplay of genetic and environmental factors and the impact of each factor varies. The DD etiology might include a major- and a minor-impact component. According to our hypothesis, there are “stress-related DD phenotypes”, namely phenotypes where key HPA axis genes may interact in gene networks with DD susceptibility genes, and the major contributing and triggering factor is stress caused by the constant difficulties encountered and the struggles of DD individuals in everyday life and especially in the school environment. A gene network bioinformatic analysis of HPA axis genes and dyslexia susceptibility genes is performed and interactions between the two groups of genes are presented. This is a multidisciplinary pilot study that focuses on DD and stress and proposes an innovative procedure and protocol. In this study genetic, epigenetic and mitochondrial molecular findings are co-evaluated with EEG findings and DASS21 scores, in order to test the possible implications of the HPA axis in the coordination of a complex regulatory network of stress associated with DD.

## 2. Materials and Methods

### 2.1. Participants

The dyslexia group (DD) consisted of ten individuals (4 males, 6 females) with an average age of 21.3 years (mean + SD/21.3 + 2.1), who underwent a school-age intervention, and a control group of ten matched controls (2 males, 8 females) with an average age of 20.7 years (20.7 ± 1.2) (control). The sample participants were higher-education students, recruited from the Department of Speech & Language Therapy of the University of Ioannina, Greece (data published in [95]). The recruitment procedure regarding individuals with DD was based on their official diagnosis meeting the Diagnostic and Statistical Manual of Mental Disorders, Fifth Edition (DSM-5) [96]. All individuals who participated in this study provided written informed consent, in accordance with the Declaration of Helsinki (1975, revised 2013). They also consented to the analysis, processing and publication of the data. The study was approved by the Research Ethics and Conduct committee of the University of Ioannina, with issued protocol number 28622 (23 July 2019).

### 2.2. DASS21 Scale

DD and control group individuals were assessed with the Depression, Anxiety and Stress Scale—21 Items (DASS21) [96] for the following phenotypic traits: depression (dysphoria, hopelessness, devaluation of life, self-depreciation, lack of interest, anhedonia, inertia), anxiety (autonomic arousal, skeletal muscle effects, situational anxiety, subjective experience of anxious effect) and stress (levels of chronic non-specific arousal). The DASS21 was constructed not merely as another set of scales to measure conventionally defined emotional states, but to further advance the process of defining, understanding and measuring the ubiquitous and clinically significant emotional states usually described as depression, anxiety and stress [96,97]. The DASS21 has been standardized for the Greek population with an internal consistency reliability level of α = 0.965 [98].

### 2.3. Peripheral Blood Sample Collection, DNA and Total RNA Extraction

#### 2.3.1. Peripheral Blood Sample Collection

Peripheral blood was used as it is the most widely studied and a relatively and easily accessible tissue [99]. All participants provided peripheral blood samples, after the provision of informed consent documents, in accordance with the principles of the Helsinki Declaration [100]. Per participant, 3 mL of peripheral blood was collected, and DNA and RNA were immediately extracted.

#### 2.3.2. DNA Extraction

Genomic DNA was extracted from 1 mL whole blood with the NucleoSpin Blood Kit (Macherey-Nagel, Düren, Germany, #740951). DNA was further processed for the genotyping of SNVs of interest, namely rs1360780 (*FKBP5)*, 5-HTTLPR and rs25531 (*SCL6A4)*, as well as for the investigation of the methylation status of the *NR3C1* gene and the evaluation of the mitochondrial DNA copy number (mtDNAcn).

#### 2.3.3. Total RNA Extraction

Per participant, 2 mL samples of peripheral blood were layered on 1 mL of Lymphosep, Lymphocyte Separation Media (Biosera, Nuaille, Pays de la Loire, France, #LM-T1702), and peripheral blood mononuclear cells (PBMCs) were isolated following consecutive centrifugations (1800 rpm/20 min, 1600 rpm/10 min, 1400 rpm/10 min at 4 °C). The PBMC precipitate was resuspended in 500 μL of NucleoZol (Macherey-Nagel, Düren, Germany, #740404). The Nucleospin RNA set for Nucleozol (Macherey-Nagel, #740406) was used for total RNA extraction, following the manufacturer’s instructions. cDNA synthesis by reverse transcription was performed using 0.5 μg of total RNA, according to the standard protocol of the PrimeScript RT Reagent Kit, with the gDNA Eraser (Takara, Shiga, Japan, #RR047A).

### 2.4. Stress-Associated SNV Detection

Genotyping for the rs1360780 (T > C) SNV of *FKBP5* was performed utilizing a TaqMan assay (Thermo Assay ID: C_8852038_10, #4351379, Applied Biosystems, Foster City, CA), based on the manufacturer’s instructions, and genotypes were identified with the StepOne Software v2.3 (Applied Biosystems, Waltham, MA, USA). Analysis of the polymorphic region 5-HTTLPR, characterized by a 44 bp insertion/deletion, and the SNV rs25531 (A > G), both located in the Solute Carrier Family 6 Member 4 *(SLC6A4)* gene, was carried out by a polymerase chain reaction–restriction fragment length polymorphism (PCR-RFLP) assay. PCR primer sequences were as follows: Forward: 5′-GGCGTTGCCGCTCTGAATGC-3′ and Reverse: 5′-GAGGGACTGAGCTGGACAACCAC-3′. Cycling conditions were as previously described [101]. The expected PCR amplicons were 529 bp and 486 bp, which were therefore named the Long (L) and Short (S) allele, respectively. PCR amplicons were digested with the MspI restriction enzyme (Takara, Otsu, Japan, #1150A) for the identification of the SNV rs25531, and, depending on the presence of the L or S allele, the digestion produced a discrete pattern of fragments, as previously described [101,102]. Genotyping was not possible for two control samples due to an insufficient amount of DNA.

### 2.5. Evaluation of mRNA Expression Levels of Stress-Related Genes

mRNA levels were quantified by real-time qPCR using the CFX Manager 3.1 software (Bio-Rad, Hercules, CA, USA). For each gene of interest (*NR3C1*, *NR3C2*, *GILZ*, *FKBP5*), primers were designed according to the corresponding NCBI GenBank sequences and selected using the NCBI primer design tool (Supplementary Table S1). Reactions were performed with the Universal SYBR Green Supermix (Kapa Biosystems, Wilmington, MA, USA, KK 4602), 400 nM of each set of primers and 20 ng of template cDNA. PCR cycling conditions were 94 °C for 3 min, followed by 35 cycles of 94 °C for 45 s 59 °C for 30 s, 72 °C for 1 min, and the final step at 72 °C for 10 min. All qPCR experiments were performed in duplicate. The Ct values of the GR (*NR3C1*), MR *(NR3C2*), *GILZ (TSC22D3*) and *FKBP5* target genes were normalized to the Ct values of *β-actin* as a reference gene. Datasets were analyzed using the 2^−ΔΔCt^ method and expressed as relative gene expression [103].

### 2.6. NR3C1 Gene Methylation Profile

#### 2.6.1. Bioinformatic Analysis

The human *NR3C1* gene is composed of eight coding exons and nine alternative, untranslated first exons. Seven of them (1_D_, 1_J_, 1_E_, 1_B_, 1_F_, 1_C_, 1_H_) lie lengthwise within *NR3C1* CpG island 303, being located in the proximal promoter region. CpG island 303 was retrieved by the UCSC genome browser (genome.ucsc.edu) [104].

#### 2.6.2. Methylation Analysis

The methylation profile analysis of two *NR3C1* alternative non-coding first exons, which are suggested to act as alternative promoters [105], was performed with the Pyrosequencing CpG assay methodology. PCR primers as well as sequencing primers, specific to the two different regions, were designed with the PyroMark Assay Design Software version 2.0 (Qiagen, Venlo, The Netherlands) (Supplementary Table S2). Genomic DNA isolation was followed by the bisulfite conversion of all unmethylated cytosines to uraciles according to the manufacturer’s instructions (Qiagen, #59104). PCR amplification of bisulfite-converted DNA using a reverse biotinylated 5′ end primer was performed with the PyroMark PCR kit, according to the manufacturer’s instructions (Qiagen, #978703). Then, the methylation status of all CpG sites of each region was analyzed with PyroMark Q24 MDx technology (Qiagen, #970802). Methylated and unmethylated (bisulfite-converted) human DNA plus unmethylated (physiological) human DNA were used as internal controls in every round of the pyrosequencing reaction. The bisulfite conversion efficiency was checked using quality controls in every pyrosequencing run set. Variations in the methylation levels of various CpG sites were detected and presented in a sequence context. Two independent pyrosequencing reactions were performed for every DNA sequence tested.

### 2.7. Mitochondrial DNA Copy Number (mtDNAcn) Estimation in Blood

Blood mtDNAcn was evaluated by qPCR, using the CFX Connect Real-Time PCR Detection System (Bio-Rad). The mtDNAcn content in genomic DNA was quantified by the CFX Manager 3.1 software (Bio-Rad), using specific primers to target the nuclear DNA: *GAPDH* Forward 5′-GTGGTCTCCTCTGACTTCAACA-3′, *GAPDH* Reverse 5′-ACCACCCTGTTGCTGTAGCC-3′. In addition, specific primers were used to target the mtDNA [106]: L394 Forward 5′-CACCAGCCTAACCAGATTTC-3′, H475 Reverse 5′-GGGTTGTATTGATGAGATTAGT-3′. The PCR mixture contained 1× Universal SYBR Green Supermix (Kapa Biosystems, Cape Town, South Africa, #KK 4602), 400 nM primers and 10 ng of template genomic DNA. The PCR cycling program was the following: 95 °C for 10 min, followed by 40 cycles of 95 °C for 10 s, 60 °C for 30 s and 72 °C for 30 s. *GAPDH* was used as a reference housekeeping gene for the calculation of the relative expression values of mtDNAcn in each sample. All qPCR experiments were performed in duplicate. The cycle in which the product was detected was defined as Ct. The Ct values provided from the qPCR for the mtDNA were normalized to the Ct values of the reference gene (*GAPDH*), and the results were expressed by the 2^−ΔΔCt^ method [103].

### 2.8. EEG Recordings

EEG signals for each channel and rhythm were recorded with a wearable EEG device, namely the Emotiv EPOC+, following the calculation of the relative band power (RBP). The power and energy spectral analysis of EEG oscillations is a well-established method for the study of cognitive processes [107,108,109,110]. The methodology for data collection and analysis was described in detail in our previous study, where the contribution of EEG recordings to the audio–visual recognition of words was explored in university students with DD [78]. For the present study, the same dataset was used, and the energy of EEG wavelets was correlated to the findings of the molecular analysis for participants with and without DD. The EEG channels that were explored followed the International 10–20 System (AF3, F3, F7, FC5, T7, P7, O1, AF4, F4, F8, FC6, T8, P8 and O2), with the exception of two electrodes that were isolated and rejected due to loss of connectivity (F8 and its corresponding electrode F7) (Supplementary Figure S1, used with permission from [78]).

#### 2.8.1. EEG Data Acquisition

EEG recordings were conducted within a room designed to minimize sound and light, capturing data from each participant during the ongoing evaluation test. Before commencing, a skilled researcher briefed the participants on the experimental process. To familiarize them with the protocol and the equipment, each participant underwent a training session lasting 4–7 min. Throughout the evaluation and EEG recording, participants remained seated upright, in a relaxed state, with their eyes open. The duration of the EEG recording varied, spanning from 21 to 38 min (with an average of 28 min), contingent on individual test completion times. Recordings were halted if any participant experienced discomfort with the procedure or the device. A total of approximately 11.5 h of EEG data were amassed, comprising 5 h and 51 min from dyslexic participants and 5 h and 47 min from non-dyslexic participants. For data collection, the Emotiv EPOC was employed, a commercially available wearable EEG device. This device, known for its lifestyle applications, encompasses 14 sensors with corresponding felt pads positioned on the scalp following the International 10–20 System layout (including AF3, F3, F7, FC5, T7, P7, O1, AF4, F4, F8, FC6, T8, P8 and O2). Additionally, two electrodes were affixed to the mastoids to serve as reference channels. The EEG data were sampled at a frequency of 128 Hz, and electrode–scalp connections were established using a saline liquid solution applied to all sensor felt pads. The setup adhered to the guidelines of the EmotivPRO 3 software, with the connectivity quality routinely monitored at the beginning and throughout the recording process.

#### 2.8.2. Preprocessing

The recordings were conducted using a montage based on the interconnected mastoids. Subsequent to each recording session, the EEG signals were exported in “.edf” format and subjected to processing utilizing the MATLAB platform along with the EEGLAB toolbox. To mitigate interference from 50 Hz power line noise oscillations, a Butterworth notch filter was applied to the EEG signals. Additionally, a high-pass FIR digital filter was implemented at 0.5 Hz to eliminate low-frequency oscillations. Subsequently, a set of five equiripple FIR filters was devised. These filters were designed to permit frequencies within specified ranges while attenuating frequencies outside of these ranges. The design of these five band-pass filters (within the range of 0.5–4 Hz, 4–8 Hz, 8–12 Hz, 13–30 Hz and 30–60 Hz) was tailored to correspond to the distinct EEG rhythms, thereby striving to extract spectral features within each specific frequency sub-band of interest. The EEG frequency bands are described in Table 1. Following this filtering process, each EEG recording was divided into non-overlapping epochs of 10 s, and spectral features were subsequently derived from these individual 10-s EEG segments.

#### 2.8.3. Feature Extraction

From the preprocessed EEG recordings, the RBP was extracted as a feature. To derive the RBP, the power spectral density (PSD) of the signal within distinct frequency bands was acquired by employing the Welch method. This technique involves segmenting the signal into overlapping fragments and computing the squared magnitude of the discrete Fourier transform for each segment. By averaging these computed values, a comprehensive estimate of the PSD is generated. Subsequently, the relative proportion of PSD for each frequency band within each time epoch was computed, resulting in a feature matrix comprising five attributes for each row. To determine the relative ratio of PSD for a particular frequency band, the PSD of that band was first computed and subsequently divided by the PSD encompassing the entire frequency range of interest, specifically spanning from 0.5 Hz to 45 Hz.

### 2.9. Gene Network Analysis

GeneMANIA (http://genemania.org accessed on 21 July 2023) [111] is a tool that is used to generate hypotheses about gene functions, visualize gene function networks and analyze gene functions and interactions by integrating proteomic and genomic data from various sources. Given a query list, GeneMANIA lists genes that share properties or functional similarities with the original query. It also shows a functional relationship network, indicating relationships between the lists and curated genomic and proteomic data [111].

### 2.10. Statistical Analysis

Statistical analysis was performed using GraphPad Prism 8 (GraphPad 8.0.1 Software, San Diego, CA, USA). All data were examined for normality of distribution with the D’Agostino–Pearson and Shapiro–Wilk tests, and since all datasets did not follow a Gaussian distribution, they were then analyzed with non-parametric statistics. The DASS21 scores, mRNA levels and methylation status of DD and control individuals were compared with the non-parametric Mann–Whitney test, and molecular data were correlated with the results of the DASS21 questionnaire with Spearman’s correlation coefficient. Results are presented after correcting for and excluding outliers with the ROUT method. Data are expressed as mean ± SEM. Statistical significance was considered for *p* < 0.05 with 95% confidence intervals (CI). Correction for multiple statistical testing was performed using Hochberg’s correction by employing a free online tool available at https://multipletesting.com (accessed on 26 June 2023) [112].

## 3. Results

In the present study, all participants were assessed with the DASS21 questionnaire for depression, anxiety and stress status. Each participant provided a peripheral blood sample, which was processed for the quantification of the mRNA levels of stress-associated genes, the genotyping of stress-associated SNVs, the methylation status of *NR3C1* and mtDNAcn evaluation. All participants underwent the EEG procedure, in order to correlate the molecular findings with EEG readouts.

### 3.1. Demographic Data, DASS21 Scores and SNVs

The psychological profiles of all participants were assessed using the DASS21 scale. Specifically, the scores for depression, anxiety and stress were measured for each participant, in order to evaluate the contributing effect and potential comorbidity of such phenotypes with DD occurrence. Descriptive statistics concerning the characteristics of DD and control human samples are presented in Table 2. Demographic information, including age (years), gender (males/females, M/F) and DASS21 scores and variants of interest are presented for individuals with DD, controls and the total sample. The control and DD groups presented non-statistically significant variations in depression and anxiety scales. Each participant was genotyped for rs1360780 of *FKBP5*, the 5-HTTLPR polymorphism and rs25531 of the *SLC6A4* gene, albeit not significant. The allelic frequencies and statistical analysis are presented in Table 2.

### 3.2. Altered mRNA and Methylation Levels in NR3C1 Gene in DD

**Figure 1:**
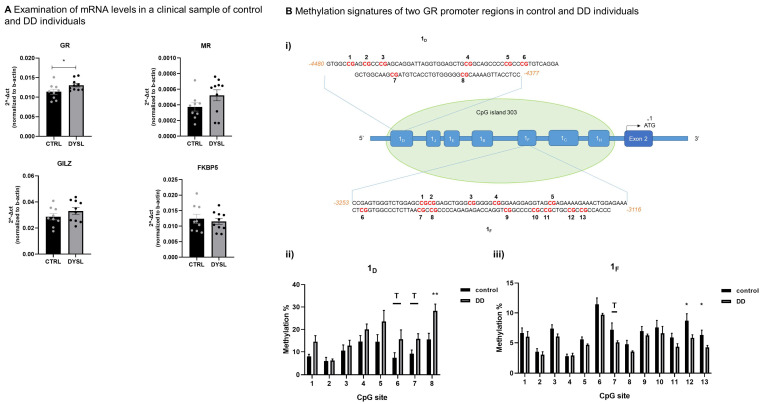
mRNA levels of stress-associated genes of interest as well as methylation status of regions 1_D_ and 1_F_ in the *NR3C1* gene promoter. (**A**) Examination of mRNA levels *NR3C1 NR3C2*, *GILZ* and *FKBP5*, DD samples vs. control samples. (**B**) (**i**) Schematic representation of the CpG island of *NR3C1* gene with individual sites for 1_D_ and 1_F_ alternative untranslated first exons. (**ii**) Methylation percentages of the 1_D_ CpG and (**iii**) 1_F_ CpG sites. Asterisk (*) indicates a *p-*value < 0.05 and a double asterisk (**) indicates a *p-*value < 0.01. Abbreviations: GR, glucocorticoid receptor; MR, mineralocorticoid receptor; *GILZ*, glucocorticoid-induced leucine zipper.

In order to study the HPA axis gene expression and methylation statuses, we analyzed the mRNA levels of the *NR3C1*, *NR3C2*, *GILZ* and *FKBP5* genes, considered key players in the neuroendocrine stress response. As presented in Figure 1A, *NR3C1* mRNA levels showed increased expression in the PBMCs of DD individuals compared to control samples (Mann–Whitney test, * *p* = 0.035, 95% CI: −0.003388 to −0.0001636), and the difference remained significant after applying Hochberg’s correction for multiple testing. To follow up on this finding, the methylation status across the *NR3C1* 5′ untranslated region (5′ UTR) was then examined (Figure 1B(i)). The 1_D_ region (Figure 1B(ii)) showed a trend (T) for higher average methylation in DD compared to control individuals. Further statistical analysis revealed a trend towards increased methylation in CpGs 6 and 7, as well as a statistically significant increase in CpG 8 (Mann–Whitney test, * *p* = 0.004), in DD compared to control individuals. In the 1_F_ region (Figure 1B(iii)), the average methylation percentage in DD samples was decreased. CpG 7 appeared hypomethylated, whereas CpGs 12 and 13 revealed a statistically significant decrease (Mann–Whitney test, * p_12_ = 0.036 and * p_13_ = 0.0304) in methylation percentages in DD compared to control samples. After applying the Hochberg correction, the differences that remained statistically significant were in CpG 8 (1_D_ region) and CpG 13 (1_F_ region). Data are presented in Supplementary Table S3.

### 3.3. Correlation of Stress-Related Molecular Changes and Behavioral Characteristics

To examine whether there was an association of the altered *NR3C1* methylation profiles with the psychological characteristics of both the DD and control individuals, we correlated the methylation percentages of specific CpG sites in the regions 1_D_ and 1_F_ with the DASS21 scores. The methylation percentage of CpG 12 located in the 1F *NR3C1* region was positively correlated with each individual’s stress scores only in DD (Spearman’s r = 0.6481 and * *p* = 0.0469) and not in control samples. Data are presented in Supplementary Table S4 and Supplementary Table S5, respectively. The statistical analysis of the mRNA levels of the stress-associated genes *NR3C1*, *NR3C2*, *GILZ* and *FKBP5* did not reveal significant correlations (Supplementary Table S6).

### 3.4. Correlation of mRNA Levels with EEG Recordings

We correlated the individual EEG PSD from different brain areas with the mRNA levels of important stress mediators, using Spearman’s correlation (Table 3). The two groups studied showed associations in different brain areas. In DD individuals, the statistically significant associations were in the lower frequencies (delta and theta), whereas, in the control group, they were in the higher frequencies (alpha and beta) (Table 3). A schematic representation of the EEG channels that were correlated with stress-associated genes is provided in Supplementary Figure S2.

### 3.5. NR3C1 Changes Do Not Affect Mitochondrial DNA Copy Number (mtDNAcn)

*NR3C1* has been found to play its biphasic roles by affecting mitochondrial function. Specifically, low doses of glucocorticoids functioning via GR in mitochondria have neuroprotective effects, while high doses exert neurotoxic effects [40,41]. In order to evaluate the GR impact on mitochondrial numbers, we assessed the mtDNAcn, a proxy for the assessment of mitochondrial numbers, in DD patients compared to the control group. We found no significant differences between the two groups (Mann–Whitney test, *p* = 0.3154, Supplementary Figure S3), suggesting that the *NR3C1*-observed changes did not impact mitochondrial abundance.

### 3.6. Gene Network Analysis

**Figure 2:**
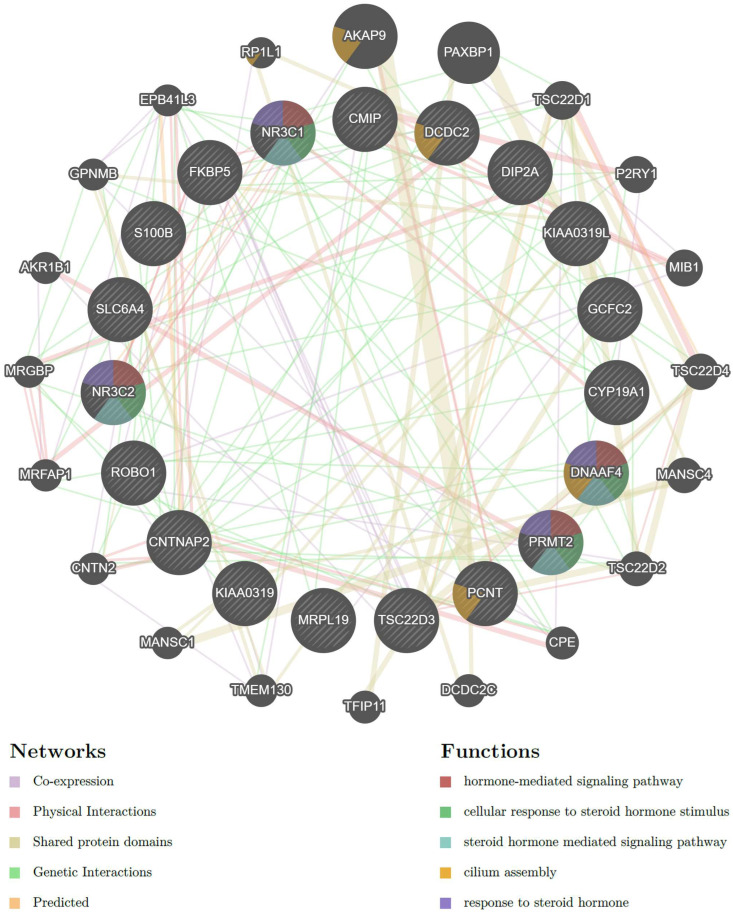
Gene interaction network of dyslexia and stress-associated genes. Target genesare shown in the inner circle, while genes associated with the query genes are shown in the outer one. The left panel presents the different types of interactions with respective color coding, depicted with lines connecting genes in the network: physical interactions (pink), predicted (orange), co-expression (purple), genetic interaction (green) and shared protein domains (yellow). The right panel presents gene functions, represented with colored strips within the respective genes: hormone-mediated signaling pathway (red), cellular response to steroid hormone stimulus (green) and steroid hormone-mediated signaling pathway (blue), cilium assembly (orange) and response to steroid hormone (purple).

The gene network analysis included 19 target genes: genes associated with reading, speech and language skills (*CMIP*, *CNTNAP2*, *CYP19A1*, *DCDC2*, *DIP2A*, *DYX1C1*, *GCFC2*, *KIAA0319*, *KIAA0319L*, *MRLP19*, *PCNT*, *PRMT2*, *S100B*, *ROBO1*) [113,114,115] along with stress-associated genes (*NR3C1*, *NR3C2*, *GILZ*, *FKBP5*, *SLC6A4*). The resulting gene interaction network predicted 20 more genes that were linked to the 19 target genes (Figure 2). Table 4 lists the specific interactions of the stress-related genes with other genes in the network.

## 4. Discussion

DD is a complex neurodevelopmental condition. As in all multifactorial phenotypes, individual genetic variations and epigenetic differences should be considered in deciphering the underlying mechanisms. The etiological heterogeneity that characterizes these conditions is due to genetic (gene × gene interactions, G × G) and/or environmental factors (G × E) interactions but the relative impact of each factor is not always clear. The understanding of the basis of phenotype variation may aid classification and facilitate targeted interventions.

Stress is an important factor for the occurrence and progression of multifactorial phenotypes, and stress pathways are often included in relevant studies. The HPA axis includes important molecular components, such as the GR and MR receptors, which mediate the neuroendocrine response to stress and have been found altered in complex neurodevelopmental phenotypes, including DD, highlighting the existence of an inadequate stress response [29,93,116,117]. In phenotypes that are stress-related, a targeted intervention may prove effective [118,119,120]. A gene network analysis of stress- and dyslexia-associated genes revealed that the majority of the stress-associated genes studied showed some form of interaction with at least one of the known dyslexia-associated genes, supporting the notion that a number of DD susceptibility genes may respond to stress and the existence of a putative DD–stress phenotype [17,29,93] (Table 4).

This study focused on genetic and epigenetic parameters coupled with psychological assessments and brain activity. We investigated the expression levels of important HPA axis stress mediators (*NR3C1*, *NR3C2*, *GILZ* and *FKBP5)* and the methylation status of the *NR3C1* promoter, coupled with DASS21 scores and EEG recordings in the two groups (DD–controls). The increased *NR3C1* mRNA levels in the PBMCs of DD individuals compared to control samples (Mann–Whitney test, * *p* = 0.035) reinforces the epigenetically mediated effects of stress on the DD phenotype.

The DD and control groups showed no statistically significant differences in DASS21 scores, but alterations were observed in *NR3C1* mRNA expression levels. This result was possibly due to the nature of the sample. The fact that the DD individuals in this study were students in higher-education institutions suggests that the longitudinal support received during their childhood was rather effective. Such an intervention may potentially contribute to the acquisition of mechanisms and strategies adequate for academic achievement, thus strengthening their stress management and positively affecting their general psycho-emotional and social development [121]. Therefore, stress effects are not reflected in the psychological evaluation and remain undetected with the current assessment techniques [122]. Exposure to adversities during early childhood could have a lifelong impact on HPA axis functioning and therefore the molecular approach probably permits a more sensitive analysis [123,124,125]. The alterations observed in the DD group vs. control group in *NR3C1* mRNA expression levels could imply an inadequate stress response, mainly reflecting the impact of the memory of stressful DD experiences, characterized by struggles and failure in the school environment, which was not apparent in the DASS21.

The epigenetic analysis of the *NR3C1* promoter region focused on the study of eight CpG sites within the 1_D_ region and thirteen CpG sites within the 1_F_ region and revealed alterations in methylation in CpG site 8 of 1_D_ and CpG 13 of the 1_F_ region. CpG site 8 of 1_D_ showed higher, statistically significant, methylation levels, and CpG 13 of the 1_F_ region showed lower, statistically significant, methylation levels in DD compared to the control group. Epigenetic modifications of the promoter region of the *NR3C1* gene, including alternative non-translated first exons 1_D_ and 1_F_, have been associated with GR expression in cases of stressful experiences and complex neuropsychiatric conditions [126,127,128,129,130]. Specifically, alterations in the methylation levels of CpGs across 1_D_ and 1_F_ are reported in the literature in cases of stressful life experiences, often related to different stressors and neuropsychiatric conditions [126,129,130,131,132,133,134,135]. The reported findings are not always reproducible because of the different conditions and pathologies studied and the variety of stressors, tissue samples and detection methods [127,131,136,137,138]. Although the reported results are sometimes contradictory, a number of studies are in agreement with this study’s results [128,132,139,140,141].

In complex heterogeneous conditions, such as DD, the variable phenotypes might be due to the incomplete penetrance and variable expressivity of the genes involved. Genomic regulation, expression and phenotypic variation are the result of the interplay of genetic and epigenetic interactions. Some of the key players in these procedures are inter- and intra-individual genomic variations, the genomic architecture, G × G interactions, the binding sites of different transcription factors and the recognition sites of epigenetic marks [142,143]. A variety of binding sites for transcription factors exist across the *NR3C1* first exons [31,128,144,145,146]. Genetic variants located in binding sites may affect the methylation of different CpG sites and gene expression and contribute to phenotypic variation [147,148,149]. Moreover, the correlation between gene expression and CpG methylation can be positive or negative and depends on the CpG site location, even if it concerns neighboring CpG sites [150].

No differences concerning mtDNAcn were observed in DD individuals compared to controls in this study sample. This was probably due to the sample consisting of young adults who were able to manage the DD difficulties. According to unpublished data from our research group, different results were obtained in a study group with a different demographic composition.

Alterations in the EEG-recorded rhythms have been extensively described both in DD and stress [151,152]. In this context, unique DD patterns were identified in a sample of young adults, by analyzing EEG recordings [78]. The pairing between EEG energy and the mRNA levels of stress-related genes indicated a shift in the brain activity towards low-frequency rhythms in the sensory and association brain areas (occipital, parietal and temporal) in both hemispheres, correlated to the mRNA levels of stress-related genes (*NR3C1*, *GILZ*, *FKBP5*). This energy increase of lower frequencies and energy decrease of higher frequencies was observed both in the DD and control groups and aligned with previous EEG findings related to stress [153]. However, a systematic review of EEG alterations in DD revealed that this pattern was also typical for dyslexia [154], despite the broad spectrum of alterations described during specific reading tasks [85,155,156]. The observed overlap in EEG findings between different disorders underscores the common features and suggests that caution is needed in interpreting the study results [153]. The prevalence of slow waves in sensory and association brain areas could be attributed to cognitive processing driven by subcortical regions responsible for emotions and motivation [153,157]. Our results also indicate that the energy decrease of fast waves was more pronounced in the DD group than the control group. This reduced EEG energy for fast waves may be correlated with low attentional control and reduced mental focus in receiving and processing sensory information, as previously described [158], which could also be related to language processing difficulties. It is important, however, to note that significant correlations between EEG and mRNA levels for the target genes were not observed in the same brain area and frequency band combination between the control and DD groups. This discrepancy can be attributed to the differential network interactions that have been described in adults with DD [159]. Further EEG studies on brain connectivity in larger cohorts of well-characterized DD samples might illustrate a putative underlying network disruption, characteristic of a stress-related DD phenotype.

The DASS21 and EEG findings revealed a positive correlation of the methylation percentage in CpG 12 of the 1_F_ region with the individual stress scores in the DD group. Moreover, changes in the expression patterns of the stress-related genes were also paired with a shift in brain activity towards low-frequency EEG rhythms in the affected sensory brain areas, which was possibly indicative of *NR3C1-*mediated HPA axis dysfunction in DD patients, which may act as a predisposing factor. The imbalance of the MR and GR populations’ distribution in typical stress centers, where they are both co-localized, could imply a putative stress-related DD phenotype.

Stress, and especially stress due to the constant difficulties and frustration associated with the school environment, may be an important factor in DD that can compromise negative feedback mechanisms, thereby leading to the persistent activation of the HPA axis [16,17]. In this study, the hypothesis that there is a stress-related DD phenotype was tested using a multidisciplinary approach composed of different modalities. In this context, the clinical diagnostic criteria of established methods, such as EEG and the DASS21 psychological assessment scale, were co-evaluated with genetic and epigenetic data, backed by bioinformatic gene network analysis data, in order to contribute to the literature an innovative model that may pave the way for the characterization of stress-specific molecular biomarkers, important for an individualized approach in DD diagnosis and carefully designed interventions. The putatively reversible nature of epigenetic alterations makes them a good candidate for stress-specific targeted interventions that aim to address environmental triggers. In the context of the hypothesis that there are DD phenotypes highly influenced by stress, this pilot study provides data and a demonstration of a new multidisciplinary approach that combines established clinical methods with EEG recordings and molecular findings of stress-associated genes.

## 5. Limitations

This was a pilot study with a rather small number of participants. Among the other limitations of the study, we highlight the heterogeneity of the sample population regarding the male:female ratio in the DD and control groups, and the fact that DD participants underwent an individualized intervention during their primary school years that targeted only the reading and writing difficulties and not stress management. Meanwhile, the reading, speech and language skills were not evaluated during the procedure; their academic performance was also not considered. It must be noted, however, that all participants were university students at the same institution, and they were allowed to take an oral or a written and oral exam, which may have alleviated some of the stress and anxiety. It must also be emphasized that this study was a pilot one, with the primary goal to present a new multidisciplinary transdiagnostic model and approach that includes stress in the occurrence of DD phenotypes, which hopefully will foster scientific interactions. Studies in substantially larger sample sizes, with a balanced sex distribution and carefully defined phenotypes (e.g., type of dyslexia and intervention), taking into account different types of educational systems and exams, as well as cohorts of people who have received not only speech and language interventions but also psychotherapy for stress, are required. Moreover, standardized genome-wide studies will be valuable in the understanding of the underlying mechanisms and the role and impact of stress in DD.

## Figures and Tables

**Table 1:** Frequency bands.

Frequency Range	Frequency Band
0.5–4 Hz	Delta
4–8 Hz	Theta
8–12 Hz	Alpha
12–20 Hz	Beta1
20–30 Hz	Beta2
30–45 Hz	Gamma

**Table 2:** Demographic characteristics regarding the DD and control individuals. DASS21 questionnaire scores and statistical values of the depression, anxiety and stress parameters examined (Mann–Whitney tests) and allelic frequencies of the genotyped variants of interest, namely *FKBP5* rs1360780, *SCL6A4* 5-HTTLPR and *SLC6A4* rs25531, for both the DD and control individuals. Abbreviations: DASS21, Depression, Anxiety and Stress Scale—21 Items; *FKBP5*, FK506 Binding Protein 5; *SLC6A4*, Solute Carrier Family 6 Member 4.

Demographic Information		Dyslexia (*n* = 10)	Control (*n* = 10)	Total (*n* = 20)	*p*-Value
Age (years), mean ± SD		21.3 ± 2.1	20.7 ± 1.2	21 ± 1.7	0.6168
Sex (M/F)		4/6	2/8	6/14	-
DASS21 Scores		Dyslexia (*n* = 10)	Control (*n* = 10)	Total (*n* = 20)	*p*-Value
Depression, mean ± SD		17 ± 10.7	13.8 ± 11.8	15.4 ± 11.1	0.4445
Anxiety, mean ± SD		15.4 ± 12.4	8.8 ± 5.4	12.1 ± 9.9	0.2212
Stress, mean ± SD		19.4 ± 9.6	19.6 ± 12.1	19.5 ± 10.6	0.8975
Allelic Frequencies		Dyslexia (*n* = 10)	Control (*n* = 8)	Total (*n* = 18)	
*FKBP5* (rs1360780)	C	0.7	0.625	0.7	
	T	0.3	0.375	0.3	
*SLC6A4* (5-HTTLRP)	L	0.4	0.625	0.5	
	S	0.6	0.375	0.5	
*SLC6A4* (rs25531)	A	1	1	1	
	G	0	0	0	

**Table 3:** Statistically significant correlations between EEG PSD and mRNA levels of stress-associated genes. Asterisk (*) indicates a *p-*value < 0.05 and a double asterisk (**) indicates a *p-*value < 0.01.

Gene	Brain Area	Rhythm	Correlation	Spearman’s r	*p*-Value
Control					
*NR3C1*	T7	Beta 1	Negative	−0.783	<0.05 *
	T7	Beta 2	Negative	−0.700	<0.05 *
	P7	Alpha 2	Negative	-0.750	<0.05 *
	O2	Delta	Positive	0.821	<0.05 *
*GILZ*	P7	Alpha 2	Negative	−0.917	<0.01 **
	P8	Theta	Positive	0.786	<0.05 *
	O1	Beta 2	Negative	−0.827	<0.05 *
*FKBP5*	P7	Beta 2	Negative	−0.883	<0.01 **
	P8	Alpha 1	Negative	−0.929	<0.01 **
DD					
*NR3C1*	P8	Theta	Positive	0.883	<0.01 **
	P8	Beta 1	Negative	−0.717	<0.05 *
*GILZ*	O1	Theta	Positive	0.881	<0.01 **
	O1	Delta	Positive	0.786	<0.05 *
*FKBP5*	T8	Delta	Positive	0.717	<0.05 *

**Table 4:** Interacting genes and networks of the studied stress-related genes according to the GeneMANIA gene interaction network. Genes associated with reading, speech and language skills are in bold.

Gene	Interacting Gene	Networks
***NR3C1***	*NR3C2*	Physical Interactions, Shared Protein Domains
	***CNTNAP2***	Genetic Interactions
	***PCNT***	Genetic Interactions
	***CYP19A1***	Physical Interactions, Genetic Interactions
	***DIP2A***	Genetic Interactions
	***CMIP***	Genetic Interactions
	*FKBP5*	Physical Interactions
	***S100B***	Genetic Interactions
	*PAXBP1*	Genetic Interactions
	*AKAP9*	Co-Expression
	*EPB41L3*	Genetic Interactions
	*CNTN2*	Genetic Interactions
	*CPE*	Genetic Interactions
***NR3C2***	*NR3C1*	Physical Interactions, Shared Protein Domains
	*FKBP5*	Physical Interactions
	*SLC6A4*	Co-Expression
	*MIB1*	Genetic Interactions
	*CNTN2*	Co-Expression
	*CPE*	Co-Expression
***FKBP5***	*NR3C1*	Physical Interactions
	*NR3C2*	Physical Interactions
	***ROBO1***	Co-Expression
	*GILZ*	Co-Expression
	*TSC22D1*	Co-Expression, Genetic Interactions
	***CNTNAP2***	Genetic Interactions
	***PRMT2***	Genetic Interactions
	*P2RY1*	Genetic Interactions
***SLC6A4***	*NR3C2*	Co-Expression
	***PRMT2***	Co-Expression
	***CNTNAP2***	Genetic Interactions
	*MRFAP1*	Genetic Interactions
	*CNTN2*	Genetic Interactions
***GILZ***	*FKBP5*	Co-Expression
	*AKR1B1*	Co-Expression
	*EPB41L3*	Co-Expression
	***ROBO1***	Genetic Interactions
	*TSC22D1*	Genetic Interactions, Shared Protein Domains
	*TSC22D2*	Physical Interactions, Shared Protein Domains
	*TSC22D4*	Physical Interactions, Shared Protein Domains

## Data Availability

Raw data are available from the corresponding author upon request due to privacy/ethical restrictions.

## Supplementary Materials

The following supporting information can be downloaded at: https://www.mdpi.com/article/10.3390/brainsci14020139/s1, Figure S1: Regions of interest according to the electrode sites; Figure S2: Schematic presentation of EEG channels. Channels where EEG PSD is associated with mRNA levels of stress mediators are marked (orange—*NR3C1*, dark blue—*GILZ*, light blue—*FKBP5*). A) EEG channels that showed correlation in the DD group, B) EEG channels that showed correlation in the control group; Figure S3: mtDNAcn for the DD individuals compared to control (Mann–Whitney, *p*-value = 0.3154); Table S1: RT-qPCR primers for the genes identified; Table S2: Pyrosequencing primers for the CpG 303 in *NR3C1* promoter region; Table S3: Statistical analysis of individual CG sites in 1_D_ and 1_F_ regions; Table S4: Spearman’s correlations for the DASS21 scale and methylation percentages for 1_F_ region for DD individuals; Table S5: Spearman’s correlations for the DASS21 scale and methylation percentages for 1_F_ region for control individuals; Table S6: Spearman’s correlations for the mRNA levels of stress-associated genes and DASS21 scores for DD and control individuals.
