# Supplementary material for: Developmental Dyslexia: Insights from EEG-Based Findings and Molecular Signatures—A Pilot Study
Source: Brain Sci. 2024 Jan 28;14(2):139. doi: 10.3390/brainsci14020139 (PMC10887023; doi:10.3390/brainsci14020139)
Supplement: Supplementary file 1 [file brainsci-14-00139-s001.zip › brainsci-2762619-supplementary.pdf]

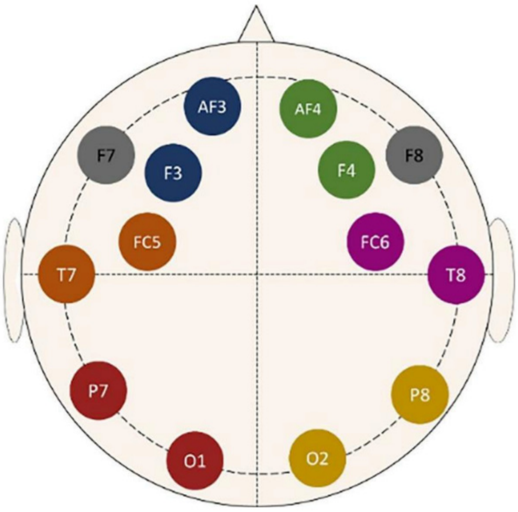

Figure S1: Regions of interest according to the electrode sites.

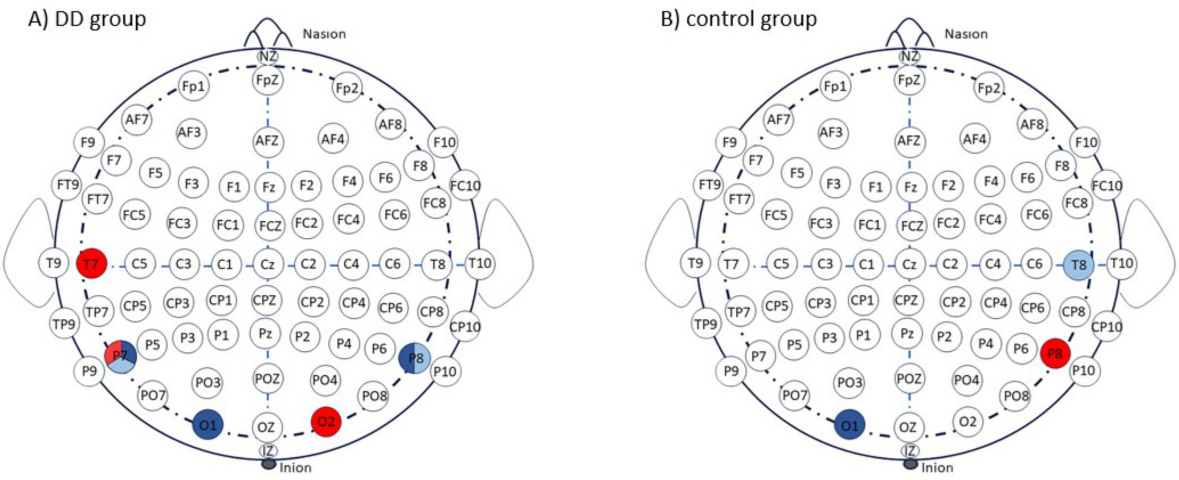

Figure S2: Schematic presentation of EEG channels. Channels where EEG PSD is associated with mRNA levels of stress mediators are marked (orange—*NR3C1*, dark blue—*GILZ*, light blue—*FKBP5*). A) EEG channels that showed correlation in the DD group, B) EEG channels that showed correlation in the control group.

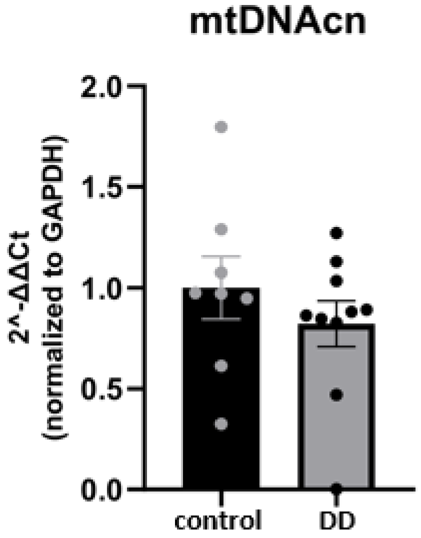

Figure S3: mtDNAcn for the DD individuals compared to control (Mann–Whitney, *p*-value = 0.3154).

Table S1. RT-qPCR primers for the genes identified

**Gene**	**Forward (5’-3’)**	**Reverse (5’-3’)**	**Amplicon Size (bp)**
β-actin	TTGCTGACAGGATGCAGAAG	ACATCTGCTGGAAGGTGGAC	141
GR (*NR3C1*)	TGAAATGGGCAAAGGCGATACC	GGTCATACATGCAGGGTAGAGT	201
MR (*NR3C2*)	TGGCAGAGGTTCTACCAACTGAC	GCAGCTGGTCGCTGATGATCTC	154
GILZ (*TSC22D3*)	CAAGATTGAGCAGGCCATGGA	TGGCTCTTCAGGGCTCAGC	199
FKBP5	GGAGAACCAAACGGAAAGGAG	CACAGTGAATGCCACATCTCTG	122

Table S2. Pyrosequencing primers for the CpG 303 in NR3C1 promoter region

Gene	CpG island	Region	Sequence	Primer
		1_D	5' ATGTTAGAGTAGGGGGAGT 3'	Forward Pyrosequencing
			5' ACCAACCTTCAACTCCCTACAAATATTCC 3'	Reverse 5' biotin labeled
NR3C1	CpG 303		5' GGTGAGAGAGTGGTT 3'	Pyrosequencing
		1_F	5' GATGAGGTGGTGGGGGATTT 3'	Forward Pyrosequencing
			5' CCCCCAACTCCCCAAAAA 3'	Reverse 5' biotin labeled
			5'GTTTTAGAGTGGGTTTGGA 3'	Pyrosequencing

Table S3. Statistical analysis of individual CG sites in 1D and 1F regions

	1_D CpG Site 1	2	3	4	5	6	7	**8**	Average Methylation
Mann-Whitney test, *p-value*	0,1039	0,2615	0,5299	0,1274	0,2109	0,065	0,0572	**0,004**	0,0650

	1_F CpG Site 1	2	3	4	5	6	7	8	9	10	11	**12**	**13**	Average Methylation
Mann-Whitney test, *p-value*	0,496	0,3846	0,1883	0,4722	0,0828	0,1139	0,0629	0,1893	0,7781	0,395	0,1024	**0,036**	**0,0304**	0,1269

Table S4. Spearman's correlations for the DASS21 scale and methylation percentages for 1_F region for DD individuals

	Depression Vs 1F CpG Site Methylation % (DD)												
	1	2	3	4	5	6	7	8	9	10	11	12	13
Spearman's r	-0,5569	-0,01258	-0,4535	0,1530	-0,2456	-0,5586	0,1901	0,1456	-0,1501	0,07143	0,07716	0,3108	0,09628
p value	0,0979	0,9758	0,1876	0,6716	0,4894	0,0962	0,5919	0,6944	0,6751	0,8443	0,8322	0,3765	0,7808
	Anxiety V_s 1_F CpG Site Methylation % (DD)												
	1	2	3	4	5	6	7	8	9	10	11	12	13
Spearman's r	-0,1451	-0,05049	-0,2368	-0,1439	-0,07556	0,09497	-0,01875	0,3386	-0,2586	0,3333	-0,2384	0,1790	0,1558
p value	0,6871	0,8908	0,5030	0,6893	0,8415	0,7930	0,9615	0,3381	0,4673	0,3437	0,5031	0,6171	0,6644
	Stress V_s 1_F CpG Site Methylation % (DD)												
	1	2	3	4	5	6	7	8	9	10	11	12	13
Spearman's r	-0,4537	0,1199	-0,3427	0,2014	0,2825	-0,1456	0,4657	-0,1594	-0,4223	-0,1620	0,2941	**0,6481**	0,5172
p value	0,1873	0,7399	0,3272	0,5744	0,4287	0,6860	0,1755	0,6599	0,2255	0,6537	0,4043	**0,0469**	0,1274

Table S5. Spearman’s correlations for the DASS21 scale and methylation percentages for 1F region for control individuals

	Depression Vs 1F CpG Site Methylation % (control)												
	1	2	3	4	5	6	7	8	9	10	11	12	13
Spearman’s r	-0,1482	0,3232	-0,5583	-0,1540	-0,07927	-0,05455	0,1091	0,01212	-0,3030	-0,1084	0,4485	0,01212	-0,3720
p value	0,7363	0,4282	0,1552	0,7167	0,8589	0,9034	0,8024	0,9835	0,4611	0,8010	0,2649	0,9835	0,3575

	Anxiety V_s 1_F CpG Site Methylation % (control)												
	1	2	3	4	5	6	7	8	9	10	11	12	13
Spearman’s r	-0,1595	-0,1515	-0,3415	-0,3190	-0,3818	-0,09036	-0,1145	-0,07229	-0,3855	0,2635	0,09639	-0,4096	-0,08485
p value	0,7057	0,7173	0,3984	0,4440	0,3484	0,8328	0,7857	0,8659	0,3429	0,5239	0,8229	0,3088	0,8395

	Stress V_s 1_F CpG Site Methylation % (control)												
	1	2	3	4	5	6	7	8	9	10	11	12	13
Spearman’s r	-0,1708	0,1446	-0,5940	-0,2029	0,000	0,1916	0,07186	-0,05988	-0,2156	-0,04762	0,1317	-0,2156	-0,4699
p value	0,7015	0,7353	0,1313	0,6452	>0,9999	0,6466	0,8716	0,8968	0,6085	0,9349	0,7605	0,6064	0,2440

Table S6. Spearman's correlations for the mRNA levels of stress-associated genes and DASS21 scores for DD and control individuals

	Depression			
	GR	MR	GILZ	FKBP5
Spearman's r	-0,02207	0,07944	0,1126	0,2666
95% CI	-0,4827 to 0,4482	-0,4010 to 0,5256	-0,3726 to 0,5494	-0,2273 to 0,6513
p value	0,9286	0,7465	0,6463	0,2700
	Anxiety			
	GR	MR	GILZ	FKBP5
Spearman's r	0,01591	-0,06540	0,3183	0,3836
95% CI	-0,4531 to 0,4780	-0,5153 to 0,4128	-0,1730 to 0,6827	-0,09990 to 0,7205
p value	0,9485	0,7902	0,1841	0,1050
	Stress			
	GR	MR	GILZ	FKBP5
Spearman's r	-0,1908	-0,05451	-0,07036	0,1486
95% CI	-0,6029 to 0,3017	-0,5073 to 0,4218	-0,5190 to 0,4087	-0,3406 to 0,5745
p value	0,4340	0,8246	0,7747	0,5438
